# K-Wire Osteosynthesis for Arthrodesis of the Paediatric Foot Is a Good and Valid Procedure

**DOI:** 10.3390/jcm12237478

**Published:** 2023-12-03

**Authors:** Jannes Kreher, Cornelia Putz, Susanne Fackler, Sebastian Müller, Axel Horsch, Andreas Geisbüsch

**Affiliations:** 1 Orthopedics and Trauma Surgery, BGU Hospital Ludwigshafen, Ludwig Guttmann Str. 13, 67071 Ludwigshafen, Germany; 2 Department of Orthopedics, Heidelberg University Hospital, Schlierbacher Landstraße 200a, 69118 Heidelberg, Germanysusanne.fackler@kabelmail.de (S.F.);; 3 International Center for Orthopedics, ATOS Clinic Heidelberg, Bismarckstr. 9–15, 69115 Heidelberg, Germany

**Keywords:** arthrodesis, clubfoot, K-wire, osteosynthesis, paediatric, pes planovalgus

## Abstract

Background: Foot deformities in children are common, and the majority can be treated conservatively. Nevertheless, there are deformities that require surgical treatment. These include rigid clubfeet, severe forms of pes planovalgus, pes cavus and several more. We retrospectively analysed the pseudarthrosis rate of surgical treatment of foot deformities with transcutaneous K-wire osteosynthesis in neurologically healthy children and adolescents. The aim of the study was to show that the results with K-wires are comparable to those with other osteosynthesis methods in the literature. Methods: A total of 46 paediatric patients aged 6 to 17 years treated between January 2010 and December 2015 met the inclusion criteria. Depending on the diagnosis, different surgical interventions were necessary. In clubfoot and pes planovalgus, representing n = 81, 70% of the whole collective triple arthrodesis with fusion of the talonavicular, calcaneocuboid and subtalar joints or Evans osteotomy was usually performed. Radiographs were taken at least 6 months post-surgery, and bony consolidation of the subtalar, talonavicular (TN), and calcaneocuboidal (CC) joints and the metatarsal I (MT I) osteotomy were assessed. If there was no evidence of fusion at this time, it was considered non-union. Results: In total, 117 arthrodesis procedures with K-wires were performed. Overall, 110 of the arthrodesis (94%) healed, and only 7 joints (6%) showed non-union (subtalar 0%, TN 7.7%, CC 6.5% and MT I 6.7%). All non-unions occurred in subjects with clubfoot deformities. No significant risk factors were observed. Conclusion: This study replicated the good consolidation rates reported in the literature with screws, plates, intramedullary nails or staples in arthrodesis of the adolescent foot in neurologically healthy subjects and confirmed the efficacy of K-wires. The main advantages of transcutaneous K-wire treatment are easy metal removal, lower osteosynthesis material costs and less concomitant damage. Further studies, especially randomised controlled trials, are needed to further investigate this topic.

## 1. Introduction

Many foot deformities affect children and adolescents, such as clubfoot, pes planovalgus, pes cavus, pes equinus, pes calcaneus and hallux valgus [1]. The incidence of clubfoot in Central Europe, for example, is 1–2/1000 [2]; symptomatic pes plano-valgus in juveniles is reported to have an incidence of 0.1/1000 [3], and juvenile hallux valgus has an incidence of 2–4% [4].

Generally, fusion surgery should be delayed to prevent changes in growth. Conservative therapy is therefore indicated for changes that are not time critical. If conservative therapy does not lead to improvement, joint-sparing interventions are indicated. If the deformity is so severe or rigid that joint sparing is not sufficient, arthrodesis surgery must be performed. Ideally, these operations are performed after the growth plates have closed. 

Fortunately, most of these foot deformities are self-limiting or can be treated conservatively. After all conservative options have been exhausted and the deformity persists, a relapse occurs or if the deformity is rigid from onset, surgical correction is indicated at an age suitable for surgery. In cases of severe deformities, we performed operations for pes planovalgus from the age of 6 years. Bony correction of idiopathic clubfeet tended to be treated surgically at a later age. However, there are contraindications for surgery, such as an underlying disease that does not allow anaesthesia, legal guardians rejecting surgical therapy, acute (florid) or chronic infection, critical soft tissue conditions or general surgical risks. The standard treatment for clubfoot is Ponseti therapy, which has good clinical results [5], followed by soft tissue interventions. This consists of tenotomy of the Achilles tendon, with posterior release and occasional realignment between the talus and calcaneus followed by an orthopaedic brace. Only if this is not successful or a relapse occurs, bony correction is necessary. Surgical treatment depends on the extent of malposition. Multidimensional correction of bony structures and soft tissue is necessary for therapy-resistant clubfoot. Triple arthrodesis, consisting of Chopart (talonavicular and calcaneocuboid joints) and subtalar arthrodesis, has proven successful [6]. In this procedure, all components of the clubfoot (equinus, cavus, adductus, varus) are corrected. In less severe cases, Chopart arthrodesis can be sufficient.

The planovalgus foot is characterised by reduction or loss of the normal medial longitudinal arch (pes planus) by hindfoot valgus/forefoot abduction and often occurs with concomitant hindfoot equinus. All these components can be flexible or rigid. Bresnahan et al. and Blitz et al. demonstrated that early conservative therapy for paediatric pes plano-valgus leads to less long-term damage [7,8]. If there is no improvement, surgical therapy is indicated. The surgical treatment of pes planovalgus is triple arthrodesis or calcaneus lengthening osteotomies, and the most common lengthening therapy is Evans osteotomy.

A severe pes cavus should be treated with Chopart and subtalar arthrodesis and pes calcaneus with inverse Lambrinudi arthrodesis. Every surgery was performed using transcutaneous K-wires.

K-wires are inexpensive and can be easily removed during and after surgery [6]. Armstrong et al. showed that the surgical time was significantly longer for patients undergoing rigid internal fixation with a screw versus a K-wire for MT I osteotomies [9]. With the transcutaneous technique, the wires remain in the foot only for the first period of bone healing and are then removed in the postoperative outpatient treatment with pliers, sometimes under local anaesthesia, or, in rare cases, with sedation [6]. The use of K-wires is recommended for all ages.

Kim et al. demonstrated that proximal chevron metatarsal osteotomy with intramedullary screw fixation provides superior biomechanical stability to locking plate and K-wire fixations [10], raising the question of whether this approach leads to higher healing rates in bone consolidation.

Characteristics of the osteosynthesis materials in terms of costs, advantages/disadvantages and indications see Table 1.

Seitz and Carpenter followed up a total of 66 patients in the years 1961–1970 and found a pseudarthrosis rate of 9.1% following triple arthrodesis. Five of the six pseudarthroses were in the talonavicular joint, and one was in the calcaneocuboid joint. K-wires were used in 22 cases, of which two did not heal; of 36 cases without osteosynthesis, four did not heal, and in the Staples group, a total of eight patients did not develop pseudarthrosis. The follow-up treatment consisted of wearing a full leg cast for 6 weeks followed by a short leg cast for 6–8 weeks [11]. Wilson et al. observed non-union in 10.3% of triple arthrodesis cases [12]. The highest incidence of non-union occurred in the talonavicular joint, followed by the calcaneocuboid and, rarely, the subtalar joint [13].

Nagy et al. showed a very good result for Evans osteotomy in pes planus, with 17 cases without non-union. They used screws for fixation [14]. Nejib et al used K-wires for lengthening calcaneal surgeries with good clinical and radiological results. One non-union occurred in a cuneiform osteotomy out of 20 flat feet surgeries [15].

Several factors leading to non-union have been described in the literature, for example, the absence of internal fixation, poor bony contact, and early weight-bearing [13]. In particular, early weight-bearing has a substantial impact. It is assumed that the resulting vertical shear forces prevent bony healing, especially in the talonavicular joint [16]. Other risk factors include age [12], the underlying diagnosis [11], the surgical technique used for fixation and deep wound infection [17].

We postulate that our treatment with K-wires and a rigid follow-up with 6 weeks of recumbent plaster and 6 weeks of walking plaster with slowly increasing weight-bearing achieves the same or even lower pseudarthrosis and non-union rates than reported in the literature for screws, plates, intramedullary nails or staples. In addition, K-wires have advantages, such as easy metal removal during an outpatient visit after 6 weeks and low material costs.

## 2. Materials and Methods

In this retrospective study, all medical records of subjects who presented to the orthopaedic clinic for arthrodesis surgery of the foot between January 2010 and December 2015 were reviewed. This study was conducted with the approval of the local ethics committee (University of Heidelberg).

Every patient under 18 years of age who underwent a foot arthrodesis operation with K-wires for a non-neurological cause was included. Different surgeons from the foot surgery department performed the standardised procedures. In approximately 70% of the subjects, the indication for the surgical procedure was clubfoot or pes planovalgus. Demographic characteristics and postoperative complications were assessed using the subjects’ medical records. The variables included age, sex, weight, comorbidities, affected side, complications, number of K-wires. The subjects were followed up regularly for a median of 25 (6; 127) months. An X-ray follow-up appointment after 6 and at least 26 weeks (median 79 weeks; 116 ± 104 weeks after) was needed. All joints that did not demonstrate bony consolidation on X-ray after 6 months were designated non-unions.

**Figure 1:**
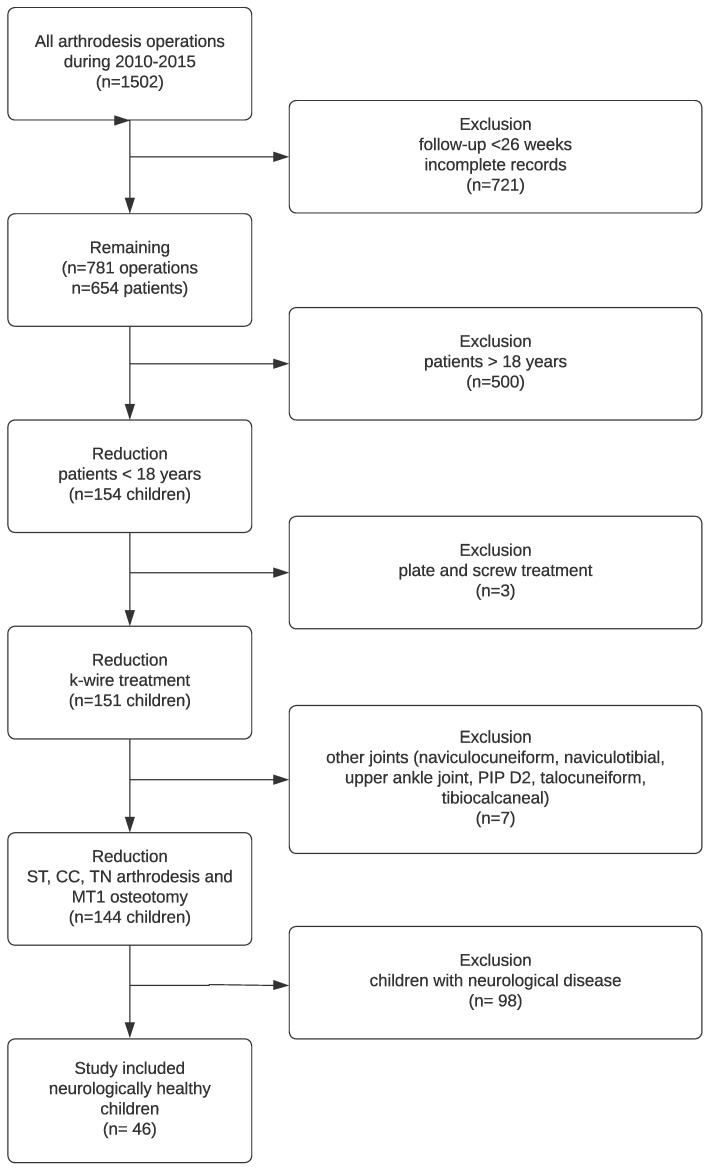
Flowchart of the study population.

As shown in the following flowchart (Figure 1), the main reason for exclusion was missing X-rays after 6 months. In total, 154 children met the follow-up criteria; in three cases, other osteosynthesis material was used, seven subjects were excluded due to joints other than the TN, CC, subtalar and MT I, and 98 patients had a neurological disease causing the deformity. Therefore, at least 46 subjects were included (Table 2).

After surgery, the young subjects continued to be treated as outpatients, and insufficient casts were changed as needed. After 6 weeks, radiographic control was performed, and the K-wires were removed. Another cast with weight bearing was applied for another 6 weeks. Thereafter, only one appointment was scheduled after 6 months for clinical and radiographic control and regular annual check-ups until the growth plates were closed.

### 2.1. Operative Procedure

**Figure 2:**
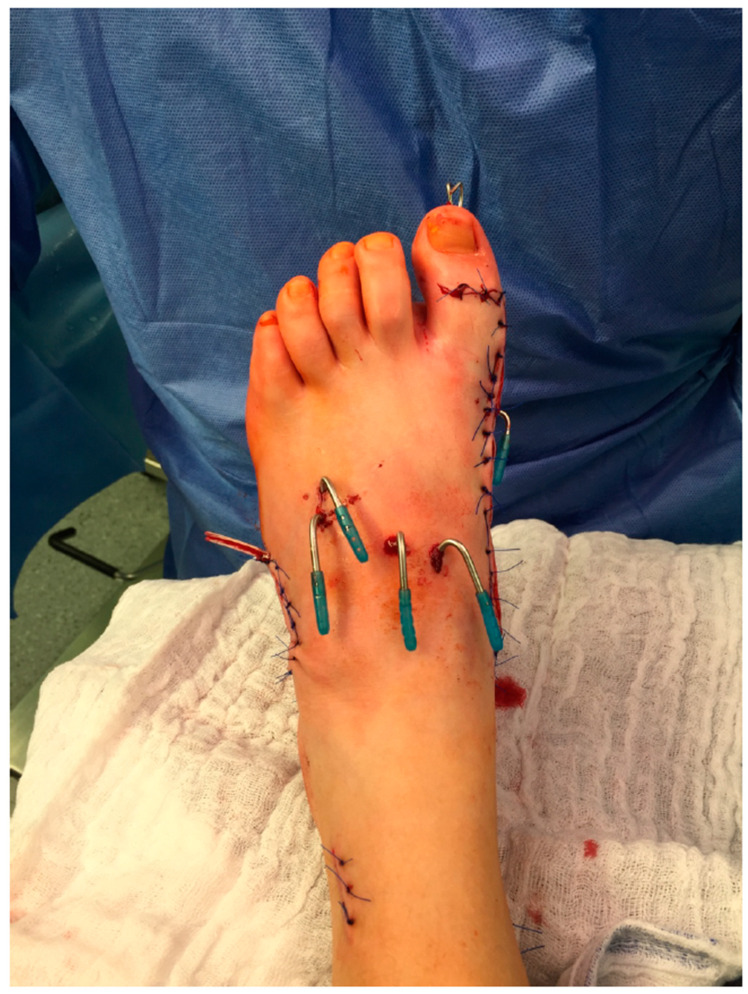
Transcutaneous K-wires for Chopart arthrodesis and Metatarsale I osteotomy plus soft tissue procedure.

It is common to combine different soft-tissue procedures (“balancing”) and bony procedures (“correction”) for the correction of multiplanar deformities. Here, we focus on bony procedures (Figure 2).

We used the surgical procedure as prescribed in Table 3 by Dreher et al. [6]:

### 2.2. Clubfoot

**Figure 3:**
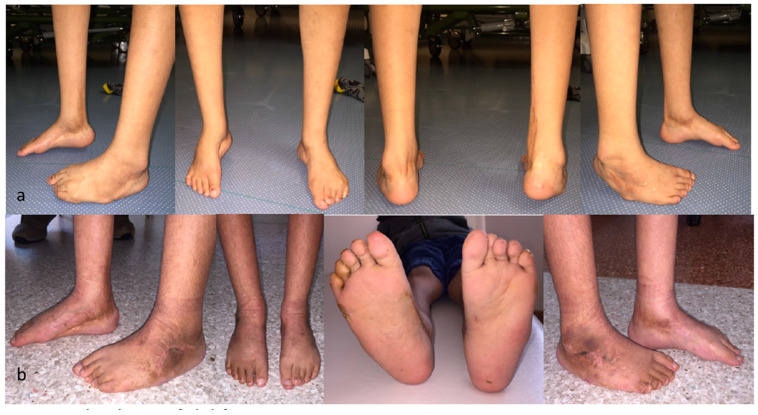
(**a**) Relapse of clubfoot. (**b**) Foot position after Chopart’s arthrodesis.

The Chopart joint was prepared, and the cartilage from the joints to be fused was subsequently removed. The foot was then redressed into its corrected position, and first, the TN joint was fixed with two K-wires. Before the calcaneocuboid joint was transfixed with two K-wires, a spreader was inserted into the subtalar space. The correct size of the lateral-based wedge was measured and harvested from the iliac crest. The iliac crest wedge was inserted, and the spreader was removed. Two more transcutaneous K-wires in parallel or in crossing technique were used to fixate the calcaneus, wedge and talus (Figure 3a,b). In milder cases, only Chopart’s arthrodesis was occasionally necessary [6,13]. 

### 2.3. Pes Cavovarus

**Figure 4:**
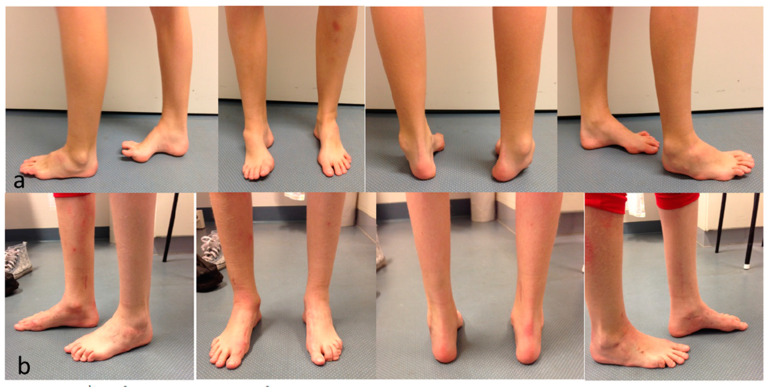
(**a**) Before correction of pes cavovarus. (**b**) Foot position after triple arthrodesis.

The first step was to release the plantar fascia (Steindler procedure) [22]. This step should be performed in all cavovarus feet because the subsequent repositioning of the foot after corrective osteotomy (Cole [23] extension osteotomy of the first metatarsal) or fusion (Chopart’s arthrodesis) of the cavus is easier. Then, the posterior tibial tendon was split into two halves and transferred through the interosseous membrane (T-SPOTT). One was used to augment the dorsiflexor, and the other was used to laterally augment the short peroneal tendon. The following step was the bony correction of the cavus and hindfoot varus with Chopart’s fusion and dorsal-based wedge resection for hindfoot reconstruction. In more severe cases, triple fusion or Lambrinudi arthrodesis [19] was performed. Finally, soft tissue procedures, claw toe correction and an extension osteotomy of the first metatarsal were performed to correct the plantar flexion deformity (Figure 4a,b) [6].

### 2.4. Pes Planovalgus

#### 2.4.1. Modified Evans Procedure

First, a calcaneal neck osteotomy perpendicular to its longitudinal axis at least 1 cm proximal to the calcaneocuboid joint was performed. Then, a transcutaneous K-wire was drilled through the cuboid bone, the calcaneocuboid joint, and the distal part of the calcaneal neck. The osteotomy was opened with a spreader until the correct position was reached, and the iliac crest bone wedge was impacted. The osteotomy and the wedge were fixed with three to four crossing K-wires [6,24].

#### 2.4.2. Calcaneocuboid Distraction-Fusion

The spreader was applied after removing cartilage from the calcaneocuboid joint. The joint was opened until the needed correction was achieved, and a prepared iliac crest bone wedge was impacted. This was followed by transfixation with three to four crossing K-wires [6,25].

#### 2.4.3. Grice Procedure

The neck of the calcaneus was exposed, and cartilage was removed from the anterior aspect of the subtalar joint. An adequate iliac crest bone wedge was prepared and inserted between the calcaneus and talus. Finally, transfixation was performed with two to four crossing K-wires [6,26].

#### 2.4.4. Triple Fusion

See the procedure for clubfoot above.

#### 2.4.5. Skewfoot

We used the surgical procedure described by Hagmann et al.: Evans osteotomy [27]:

The saw blade was inserted approximately 1.5 cm proximal from the calcaneocuboid joint without affecting the medial, dorsal, or plantar cortical shell of the calcaneus. A transcutaneous K-wire was inserted to fixate the cuboid and the distal fragment of the calcaneus to prevent dorsal deviation during osteotomy. The calcaneal osteotomy of the medial cortex was completed with a chisel. A distractor helped to determine the degree of correction needed to reduce hindfoot valgus. A trapezoid-shaped graft of the needed size was taken from the iliac crest. The graft was fixed with two to four crossing K-wires, depending on primary stability [28].

#### 2.4.6. Pes Equinus

Lambrinudi arthrodesis [19,29]: First, debridement of the sinus tarsi and removal of the bifurcate ligament were needed. Then, the subtalar, calcaneocuboidal, and talonavicular joints were exposed. For deformity correction, a bone wedge from the calcaneus and talus (25–30°) was removed. For better fitting, debridement of the talar head and navicular was performed. The cartilage of the calcaneocuboidal joint was removed. After reduction (neutral dorsiflexion and 10° foot abduction), fixation was performed with K-wires [30].

#### 2.4.7. Pes Calcaneus

Inverse Lambrinudi arthrodesis [20]: The arthrodesis consists of triple arthrodesis between the talus, calcaneus, navicular, and cuboid, in combination with inserting a bone wedge and cancellous bone between the talus and calcaneus. After this correction, a further step was necessary, and an additional bone graft was implanted between the talus and navicular bone.

#### 2.4.8. Hallux Valgus

There were different concepts for the treatment of hallux valgus depending on the severity of the deformity. Chevron and Scarf osteotomy for the correction of mild metatarsus primus varus deformities, lapidus arthrodesis (TMT I joint) for the correction of severe metatarsus primus varus deformities and Akin osteotomy for the supplementary correction of hallux valgus interphalangeus [31].

Occasionally, combinations of the operations mentioned above have been used to correct multidimensional foot deformities.

#### 2.4.9. Postoperative Treatment

**Figure 5:**
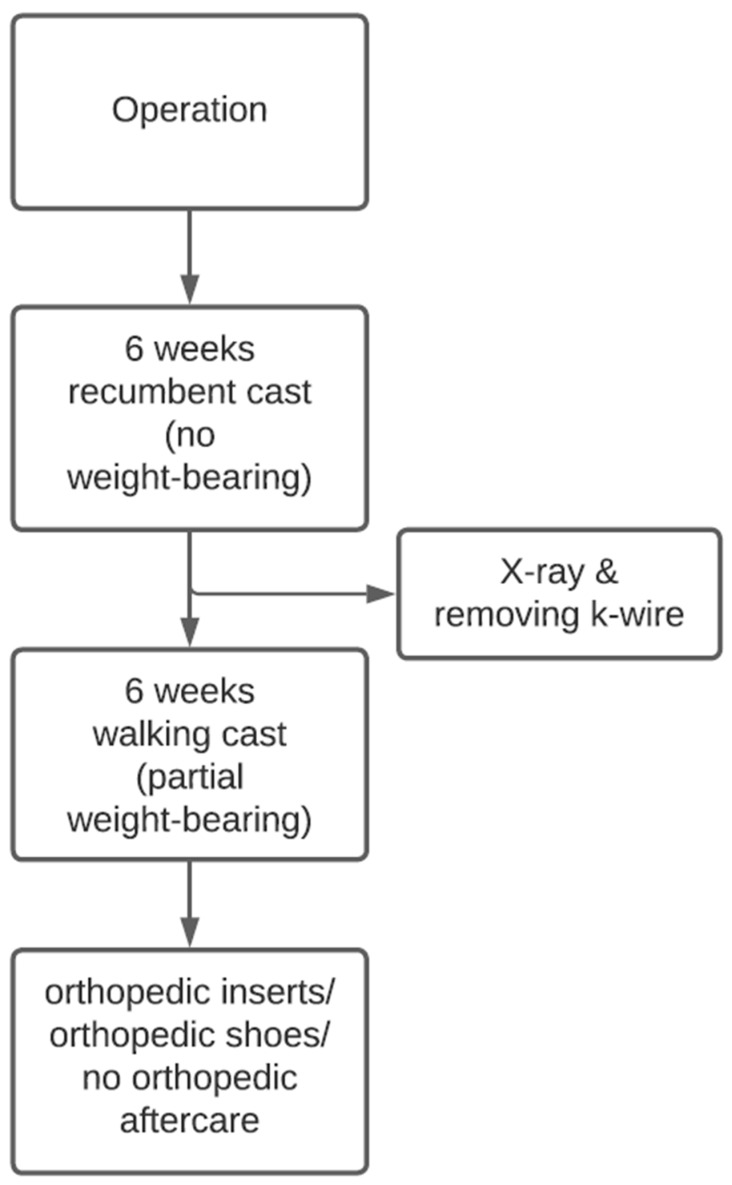
Flowchart on standardised postoperative follow-up treatment.

Standard wound checks were performed in our outpatient clinic. For this purpose, windows were cut into lower leg plaster to examine and dress the wounds. To prevent transcutaneous infections from the wires, regular wound checks should be made. Six weeks after surgery, the K-wires were removed. In some cases, local anaesthesia was necessary; wires were removed with pliers. During this appointment, the recumbent cast was changed to a walking cast, which the subjects wore for another 6 weeks. The walking cast allows partial weight-bearing, which should be increased over time with full weight-bearing occurring around the 12th postoperative week. Then, the cast was removed, and depending on the clinical findings, orthopaedic inserts or shoes were fitted in our technical orthopaedics department (Figure 5).

X-ray controls were performed in a standardised manner in two planes. Depending on the region of the arthrodesis, additional planes were sometimes included. In this manner, it was possible to reliably determine the complications of non-union. For this study, only the subtalar, talonavicular (TN), calcaneocuboidal (CC) joints and metatarsal I (MT I) osteotomy treated with K-wire osteosynthesis were examined. As described above, depending on the indication, a combination arthrodesis of different joints was necessary to achieve a good clinical result.

#### 2.4.10. Statistics

The statistical analyses were performed using IBM SPSS Statistics 28. *P* values were interpreted descriptively, and a *p* value < 0.05 was considered to indicate significance. The independence of the different factors (sex, joint, age, weight) was examined using chi-square and Mann–Whitney U tests. The statistical analysis was performed in collaboration with the Institute for Medical Biometry of the University Hospital Heidelberg.

## 3. Results

**Figure 6:**
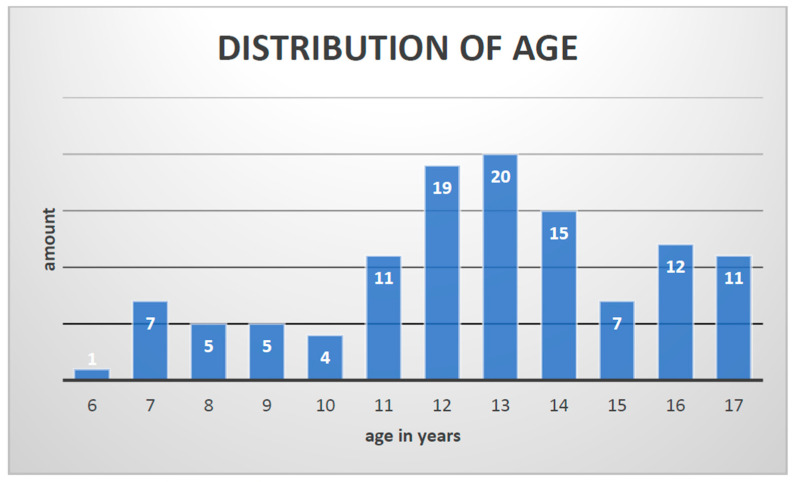
Distribution of age.

**Figure 7:**
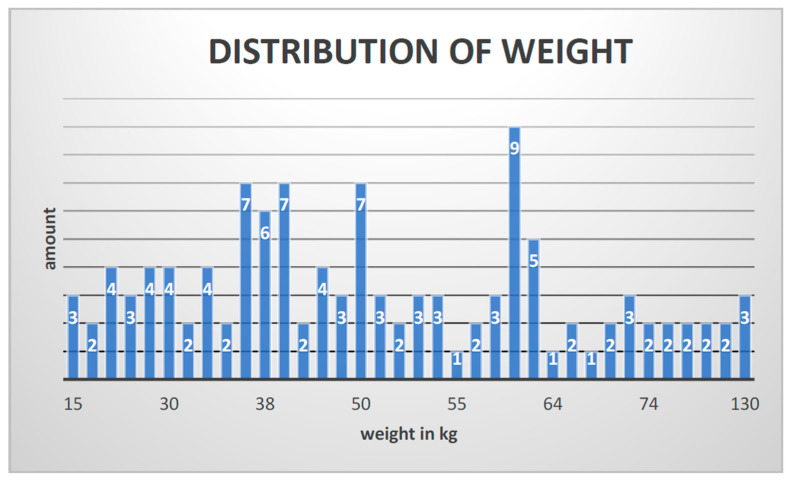
Distribution of weight.

In total, 46 subjects matched the inclusion criteria, and 117 arthrodesis procedures with K-wires were performed. The entire patient cohort was distributed as follows: 70% (81) boys and 30% (36) girls with a median age of 13 years (6–17 y; 12.68 ± 2.815) and an average weight of 50 kg (15–130 kg; 51.01 ± 22.944) (Figure 6 and Figure 7).

Neither age (*p* = 0.104) nor body weight (*p* = 0.501) was a significant risk factor for non-union.

**Figure 8:**
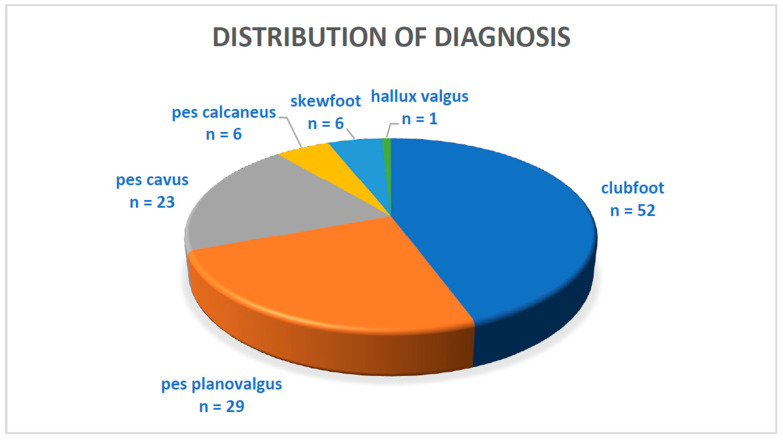
Distribution of diagnosis.

Regarding the distribution of the diagnoses (Figure 8) that led to the indication of arthrodesis, almost half of the subjects had clubfoot (52). The second most common diagnosis was pes planovalgus (29), followed by pes cavus (23), pes calcaneus (6), skewfoot (6) and hallux valgus (1).

**Figure 9:**
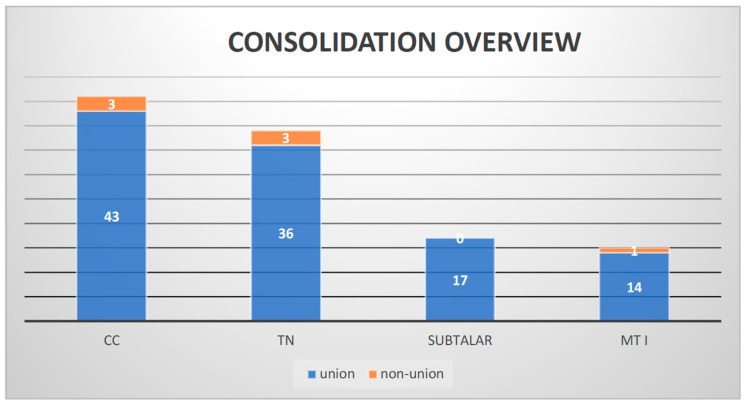
Consolidation overview.

Our follow-up showed that over 94% of the arthrodesis cases healed. Of 117 joints, only 7 showed non-union, 3 TN joints, 3 CCs and 1 MT I (Figure 9).

**Figure 10:**
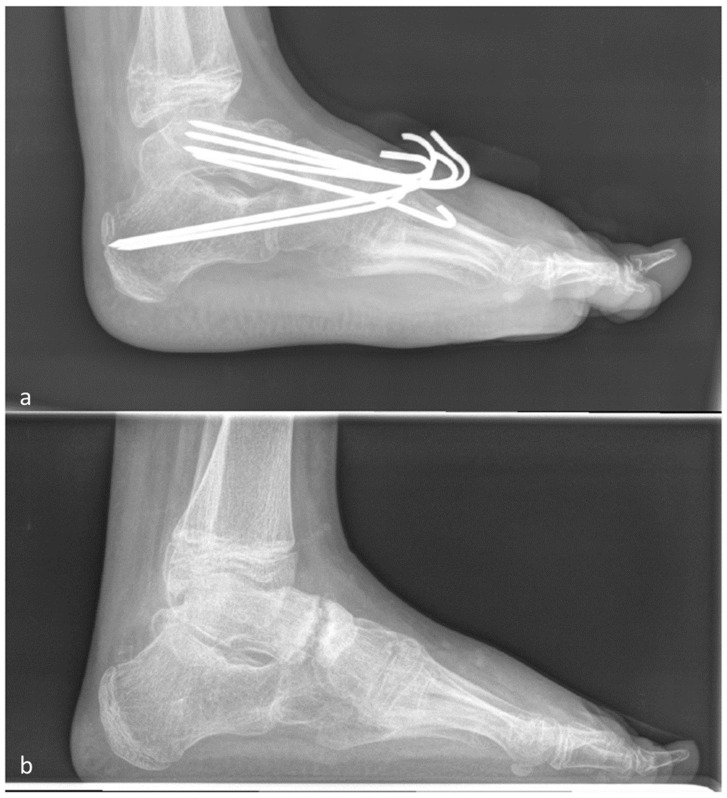
(**a**) First post-surgery X-ray after talo-navicular and CC arthrodesis with K-wires. (**b**) Talo-navicular non-union 8 months after surgery.

**Figure 11:**
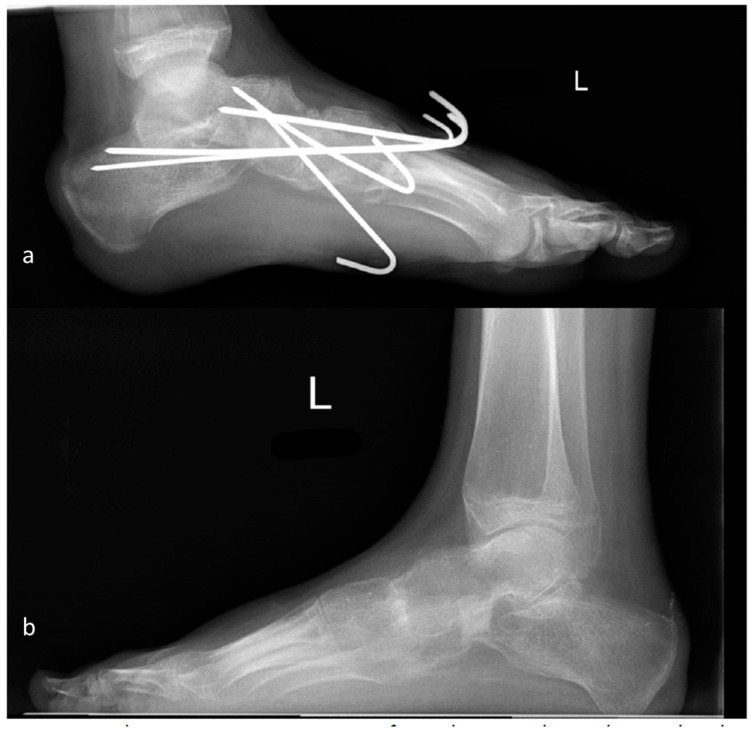
(**a**) First post-surgery X-ray after talo-navicular and CC arthrodesis with K-wires. (**b**) Talo-navicular bony consolidation 10 months after surgery.

In total, 17 subtalar arthrodesis procedures were performed, and after 26 weeks, no non-union was found. Of 39 operated talonavicular joints, 3 showed non-union (7.7%). Regarding the calcaneocuboidal joints, 3 out of 46 showed non-union (6.5%), and the surgeries at MT I demonstrated one non-union out of 15 (6.7%). No significant association between joint and non-union was observed (*p* = 0.737) (Figure 10a,b and Figure 11a,b).

Of the 46 subjects, 4 showed non-union (Table 4). The first had non-union in the TN and CC joints. The boy weighed 93 kg at age thirteen. Three revision surgeries were needed due to wound healing disorders. The second patient also had non-union in the TN and CC joints. The female patient was 10 years old, had a body weight of 37 kg and showed a wound-healing disorder that could be cured with antibiotics. The third patient, a male, 8 years old, 27 kg with a previous pantalar release and a respiratory disease, had a non-union in the TN joint. The fourth patient, a 12-year-old female weighing 35 kg, had a wound disorder that was healed by wound management and had non-union in the MT 1 and CC joints.

No significant association between poor fracture healing and a specific previous disease was observed, but all the non-union cases involved arthrodesis for clubfoot deformity. Five of the seven non-union subjects showed a wound-healing disorder in the postoperative period. Two of these needed surgery. In total, eight subjects (17%) had wound disorders. In four cases, revision was needed. The soft tissues healed properly during the procedure. Recurrence of the deformity was documented in two subjects despite correct bone healing. Further operations became necessary, one requiring Chopart arthrodesis for seven years and the other subject supramalleolar derotation approximately nine years after the initial surgery. There were no smokers or diabetes mellitus patients in our cohort.

## 4. Discussion

The purpose of this study was to investigate whether the non-union rate in arthrodesis of the foot with K-wires is comparable to screws and plates or other osteosynthesis materials. To our knowledge, this is the first study to focus on bony consolidation after K-wire osteosynthesis of various joints in the hind, mid and forefoot in children and adolescents aged 6–17 years without previous neurological disease. A total of 117 joints were re-examined in 46 children from January 2010 to December 2015. We defined non-union if no bone consolidation could be seen in the X-ray over 6 months after surgery. Due to the young patient population, standard CT diagnostics were not used.

Our results confirmed the union rates reported in the literature. Consistent with former studies, the talonavicular joint [32] had the worst outcome in our study, with a non-union rate of 7.7%. Wicks et al. demonstrated a non-union rate of 5%, which is comparable to our findings, and they also used pins and rigid postoperative treatment with 10–12 weeks of cast wearing [13]. They described 159 cases between 1971 and 2006. This retrospective study included patients younger than 16 who were operated on at the TN joint by a single surgeon with pins. Turriago et al. reported a non-union rate of 11.8% in children with cerebral palsy. They used Steinmann pins and cannulated cortical screws. Pseudarthrosis occurred in seven cases, six in the group with Steinmann pins and one in the screw group. However, no statistical significance between the occurrence of pseudarthrosis and the fixation method was found. The post-surgery treatment was a short leg cast for 6 weeks [33]. This retrospective study investigated TN arthrodesis after pes planus valgus correction between February 2002 and December 2005. A total of 59 feet were included, and the patient age ranged from 9–20 years.

Arumugam et al recently reviewed non-union rates in TN arthrodesis depending on the osteosynthesis material used: screw fixation (n = 75): 87.5% to 100%, staple fixation (n = 13): 100%, intraosseous fix system (n = 16): 100%, and K-wire fixation (n = 2): 100%. One study utilised a dorsal locking plate with two supplemented compression screws (n = 9, fusion rate = 100%), and two used a combination of screws with staples (n = 26, fusion rate = 96%) [34].

The CC joints did not heal in 6.5% of cases, consistent with the data from Moore et al., who reported a non-union rate of 7.1% for the CC joint in adults treated using screws [35]. Between 2007 and 2011, 70 patients with flat feet were retrospectively examined. The radiological follow-up was 52 weeks. The patients were transitioned to a weight-bearing Cam walker boot at 6 weeks postoperatively and allowed to gradually increase weight-bearing from 10% to 100% over the next 6 weeks with active and passive ankle movement permitted. Wicks et al. reported a slightly better outcome, with a non-union rate of 2% [13].

The 17 cases of subtalar arthrodesis all healed, consistent with previous findings; for example, Wicks et al. reported 1.3% non-union [13]. 

MT1 osteotomy showed a non-union rate of 6.7%, and the literature findings are inconsistent. Kim et al. demonstrated that proximal chevron metatarsal osteotomy with intramedullary screw fixation provides superior biomechanical stability to locking plate and K-wire fixations. The new technique using intramedullary screw fixation can offer robust fixation and may lead to better outcomes in the surgical treatment of hallux valgus [10]. Armstrong et al. found that those treated with rigid internal screw fixation for distal MT I osteotomies did not resume wearing shoes earlier, developed fewer postoperative infections, or had an increased long-term range of motion compared with the group receiving external fixation with a single K-wire. The surgical time was significantly longer for those patients undergoing rigid internal fixation with a screw (42.5 +/− 9.5 vs. 35.1 +/− 6.6 min, *p* < 0.001). Therefore, Armstrong concluded that there is no significant difference in postoperative infection, dehiscence, long-term structural correction attained, or range of motion achieved between rigid internal screws and external K-wires used to fixate distal metatarsal osteotomies [9].

In our cohort, only four cases (3.4%, two patients) needed revision due to a wound healing disorder. Superficial wound healing disorders occurred in 7.5% of patients and healed properly without surgical intervention. Wicks et al. reported a 7% wound infection rate, and Crevoisier documented a deep infection rate ranging from 0% to 2%, whereas superficial wound infection or scar dehiscence occurred in 0% to 10% of patients [13,32]. Overall, wound healing disorders seem to be a factor in bony consolidation in our study. We did not obtain a statistically significant result; however, wound-healing disorders were documented in three out of four patients with pseudarthrosis. This is consistent with the results of Galindo et al. and Martone et al. [17,36].

Regarding the gender distribution, there were twice as many boys included in the study than girls, and arthrodesis tended to heal worse in girls; however, we could not determine the reason for this, and the result was not significant. Only 3.6% of all arthrodesis in boys did not heal, while 11.1% of joints in girls showed non-union. Regarding age and weight, no significant risk factors were found.

Studies have shown that K-wires are not as stable as, for example, screws. We postulate that the posttreatment regimen of a total of 12 weeks of cast wearing, split into 6 weeks of recumbency followed by 6 weeks of walking plaster, can achieve comparable union rates. K-wire osteosynthesis coupled with rigid posttreatment leads to very good consolidation rates. K-wires also have the advantage of very uncomplicated metal removal, which can be conducted at a routine follow-up appointment after 6 weeks by pulling percutaneously inserted K-wires in an ambulant setting. This means that no foreign material remains that could irritate the musculoskeletal system, become infected or influence the surgical procedure in subsequent interventions. In addition, this procedure is much less expensive than other osteosynthesis and carries almost no periinterventional risks during removal, such as wound infection, anaesthesia-associated problems, or injury to the musculoskeletal system.

Plaster treatment ensures immobilisation, especially at a young age. When orthoses are prescribed, the patient’s compliance is not guaranteed; thus, bony consolidation is endangered by early weight-bearing.

### Limitations

Limitations of the study are the retrospective design and the inclusion of inhomogeneous interventions based on different indications and different surgeons. Unfortunately, we did not have a comparison group, which is needed to compare our results with those reported in the current literature.

Ultimately, we could not identify a significant risk factor, only a tendency in which the female sex, postoperative wound infection and the underlying disease-causing clubfoot seem to play a role.

## 5. Conclusions

We demonstrated that K-wire osteosynthesis in combination with rigid postoperative cast treatment achieves very good results, with bone healing observed in 94% of patients, and is superior to the other procedures in terms of requiring fewer operations, easier metal removal, lower costs and shorter operating time. However, the follow-up treatment includes 12 weeks of plaster and thus a long immobilisation with higher effort in follow-up care with regular checks and at least one change in plaster. This potentially leads to more expensive follow-up care and necessitates closer patient follow-up. Further randomised controlled trials are needed to confirm the results.

## Figures and Tables

**Table 1:** Modified from Dreher et al. [6], characteristics of the osteosynthesis materials.

Osteosynthesis	Advantages and Disadvantages	Cost	Indication
K-wires	Easy to use Temporary Transcutaneous infection	Low	All deformities up to 60 years Contraindication is Charcot’s foot
Staples	Additional fixation system High costs to explant	Lower-mid	Forefoot corrections Additive fixation, combined with other osteosynthesis
Cannulated screws	More stable than wires Compression option Costs to implant and explant	Medium-high	All deformities Non-unions Smokers Charcot’s foot Reduced bone quality
Locking plates	More stable than screws Polyaxial fixation expensive	High	Ankle fusion Long-distance fusion Non-unions Smokers Charcot’s foot Reduced bone quality
Nails	More stable than screws and plates expensive	High	Non-unions Ankle/pantalar fusion

**Table 2:** Sociodemographic variables.

Arthrodesis/ Osteotomy	Date of Birth	Surgery	Age (Years)	Joint	Sex	Body Weight (kg)	Foot Deformity	Side	K-Wires	K-Wire Removal	Bone Healing	Last X-ray (Weeks)	Follow Up (Weeks)	Complications	Comorbidity
N = 1	13 March 2002	26 January 2010	7	subtalar	m	25	pes planovalgus	r	2	26 February 2010	good	224	224	n	hypothyroidism
N = 2	21 October 1993	31 March 2010	16	subtalar	m	73	clubfoot	r	1	10 May 2010	good	84	84	n	n
N = 3	06 August 1998	11 January 2011	12	subtalar	m	60	clubfoot	r	2	18 February 2011	good	46	46	n	n
N = 4	31 July 1994	03 February 2011	16	subtalar	m	60	pes planovalgus	l	2	14 March 2011	good	79	130	1; 2	n
N = 5	19 December 1993	25 May 2011	17	subtalar	m	60	clubfoot	l	1	04 July 2011	good	58	58	n	n
N = 6	17 February 1998	04 October 2011	13	subtalar	f	37	clubfoot	r	2	10 November 2011	good	159	159	n	n
N = 7	29 August 1999	27 October 2011	12	subtalar	f	24	pes planovalgus	r	2	12 December 2011	good	30	30	n	n
N = 8	29 August 1999	27 October 2011	12	subtalar	f	24	pes planovalgus	l	2	12 December 2011	good	30	30	n	n
N = 9	26 May 2004	05 January 2012	7	subtalar	m	15	pes planovalgus	l	2	07 February 2012	good	420	420	3; 4; 5	n
N = 10	02 November 1998	19 September 2012	13	subtalar	m	55	pes planovalgus	l	4	05 November 2012	good	26	89	n	n
N = 11	20 November 1999	19 March 2013	13	subtalar	m	38	clubfoot	r	2	02 May 2013	good	186	186	n	n
N = 12	20 December 1997	18 June 2013	15	subtalar	m	100	clubfoot	l	2	25 July 2013	good	30	30	n	n
N = 13	05 September 2001	27 August 2013	11	subtalar	m	62	pes planovalgus	r	1	07 October 2014	good	119	119	n	n
N = 14	13 October 1997	01 July 2014	16	subtalar	m	130	pes planovalgus	r	2	14 August 2014	good	53	53	n	n
N = 15	17 June 2003	23 June 2015	12	subtalar	f	54	pes planovalgus	l	2	03 August 2015	good	310	310	n	n
N = 16	20 March 2007	11 August 2015	8	subtalar	m	30	pes calcaneus	l	3	10 September 2015	good	47	47	n	n
N = 17	20 March 2007	17 August 2015	8	subtalar	m	30	pes calcaneus	r	3	14 September 2015	good	46	46	n	n
N = 18	24 July 1992	05 January 2010	17	TN	m	50	pes cavus	l	2	15 February 2010	good	142	142	n	n
N = 19	13 March 2002	26 January 2010	7	TN	m	25	pes planovalgus	r	2	26 February 2010	good	224	224	n	hypothyroidism
N = 20	24 July 1997	18 March 2010	12	TN	m	36	clubfoot	r	3	26 April 2010	good	103	103	n	n
N = 21	21 October 1993	31 March 2010	16	TN	m	73	clubfoot	r	2	10 May 2010	good	84	84	n	n
N = 22	24 July 1992	13 April 2010	17	TN	m	51	pes cavus	r	2	26 May 2010	good	110	110	n	n
N = 23	10 July 1995	29 July 2010	15	TN	m	50	clubfoot	r	2	16 September 2010	good	127	127	n	n
N = 24	04 October 1995	20 September 2010	14	TN	m	72	clubfoot	r	2	04 November 2010	good	37	37	n	n
N = 25	11 January 2001	06 October 2010	9	TN	f	27	skewfoot	l	2	08 November 2010	good	62	107	n	n
N = 26	21 January 1997	13 October 2010	13	TN	m	93	clubfoot	r	2	13 December 2010	pseudarthrosis	147	147	1; 4	n
N = 27	26 October 1996	03 January 2011	14	TN	f	59	pes cavus	l	2	07 February 2011	good	41	63	1; 2	n
N = 28	06 August 1998	11 January 2011	12	TN	m	60	clubfoot	r	2	18 February 2011	good	46	46	n	n
N = 29	24 July 1997	02 February 2011	13	TN	m	41	clubfoot	l	3	17 March 2011	good	57	57	n	n
N = 30	31 July 1994	03 February 2011	16	TN	m	60	pes planovalgus	l	4	14 March 2011	good	79	130	1; 2	n
N = 31	24 May 1996	14 February 2011	14	TN	f	62	pes cavus	r	2	31 March 2011	good	63	63	n	n
N = 32	09 August 1995	07 March 2011	15	TN	m	38	skewfoot	l	2	21 April 2011	good	102	102	n	n
N = 33	03 January 2000	28 April 2011	11	TN	f	52	clubfoot	r	2	10 June 2011	good	507	507	3; 4; 6	n
N = 34	19 December 1993	25 May 2011	17	TN	m	60	clubfoot	l	3	04 July 2011	good	58	58	n	n
N = 35	16 September 1999	15 June 2011	11	TN	m	34	pes cavus	r	2	25 July 2011	good	56	56	n	n
N = 36	23 April 2001	20 July 2011	10	TN	f	37	clubfoot	l	4	01 September 2011	pseudarthrosis	31	31	1; 2	n
N = 37	11 June 2003	21 September 2011	8	TN	m	27	clubfoot	l	2	24 October 2011	pseudarthrosis	372	477	n	asthma
N = 38	17 February 1998	04 October 2011	13	TN	f	37	clubfoot	r	2	10 November 2011	good	159	159	n	n
N = 39	29 August 1999	27 October 2011	12	TN	f	24	pes planovalgus	r	2	12 December 2011	good	30	30	n	n
N = 40	29 August 1999	27 October 2011	12	TN	f	24	pes planovalgus	l	2	12 December 2011	good	30	30	n	n
N = 41	21 January 1997	01 December 2011	14	TN	m	101	clubfoot	l	3	05 January 2012	good	88	88	n	n
N = 42	13 February 2002	14 December 2011	9	TN	f	37	clubfoot	l	2	23 January 2012	good	210	238	n	n
N = 43	26 May 2004	05 January 2012	7	TN	m	15	pes planovalgus	l	2	07 February 2012	good	420	420	3; 4; 5	n
N = 44	02 April 1997	08 February 2012	14	TN	m	58	clubfoot	r	2	26 March 2012	good	85	85	n	n
N = 45	13 August 2000	02 May 2012	11	TN	m	40	pes calcaneus	l	2	13 June 2012	good	32	32	n	n
N = 46	02 April 1996	08 May 2012	16	TN	m	40	pes cavus	l	2	22 June 2012	good	46	98	n	n
N = 47	13 February 2002	09 May 2012	10	TN	f	43	clubfoot	r	3	18 June 2012	good	170	170	n	n
N = 48	13 August 2000	13 June 2012	11	TN	m	35	pes planovalgus	r	2	20 July 2012	good	26	26	1	n
N = 49	19 May 2000	18 December 2012	12	TN	f	44	pes cavus	r	2	01 February 2013	good	26	26	n	n
N = 50	20 November 1999	19 March 2013	13	TN	m	38	clubfoot	r	2	02 May 2013	good	186	186	n	n
N = 51	05 September 2001	27 August 2013	11	TN	m	62	pes planovalgus	r	2	07 October 2014	good	119	119	n	n
N = 52	13 October 1997	01 July 2014	16	TN	m	130	pes planovalgus	r	2	14 August 2014	good	53	53	n	n
N = 53	09 March 2000	11 February 2015	14	TN	m	64	clubfoot	l	2	23 March 2015	good	279	279	n	n
N = 54	25 August 2001	02 April 2015	13	TN	f	67	clubfoot	l	4	18 May 2015	good	189	189	n	n
N = 55	17 June 2003	23 June 2015	12	TN	f	54	pes planovalgus	l	3	03 August 2015	good	310	310	n	n
N = 56	29 June 2002	19 August 2015	13	TN	m	53	clubfoot	r	2	17 September 2015	good	198	198	n	n
N = 57	24 July 1992	05 January 2010	17	CC	m	50	pes cavus	l	2	15 February 2010	good	142	142	n	n
N = 58	13 March 2002	26 January 2010	7	CC	m	25	pes planovalgus	r	2	26 February 2010	good	224	224	n	hypothyroidism
N = 59	15 July 2003	11 March 2010	6	CC	f	20	pes planovalgus	l	4	09 April 2010	good	44	44	n	n
N = 60	24 July 1997	18 March 2010	12	CC	m	36	clubfoot	r	2	26 April 2010	good	103	103	n	n
N = 61	21 October 1993	31 March 2010	16	CC	m	73	clubfoot	r	2	10 May 2010	good	84	84	n	n
N = 62	24 July 1992	13 April 2010	17	CC	m	51	pes cavus	r	2	26 May 2010	good	110	110	n	n
N = 63	17 February 1998	18 May 2010	12	CC	f	35	clubfoot	l	2	05 July 2010	pseudarthrosis	231	231	1; 2	n
N = 64	10 July 1995	29 July 2010	15	CC	m	50	clubfoot	r	2	16 September 2010	good	127	127	n	n
N = 65	04 October 1995	20 September 2010	14	CC	m	72	clubfoot	r	2	04 November 2010	good	37	37	n	n
N = 66	11 January 2001	06 October 2010	9	CC	f	27	skewfoot	l	1	08 November 2010	good	62	107	n	n
N = 67	21 January 1997	13 October 2010	13	CC	m	93	clubfoot	r	2	13 December 2010	pseudarthrosis	147	147	1; 4	n
N = 68	26 October 1996	03 January 2011	14	CC	f	59	pes cavus	l	2	07 February 2011	good	41	63	1; 2	n
N = 69	06 August 1998	11 January 2011	12	CC	m	60	clubfoot	r	2	18 February 2011	good	46	46	n	n
N = 70	24 July 1997	02 February 2011	13	CC	m	41	clubfoot	l	2	17 March 2011	good	57	57	n	n
N = 71	31 July 1994	03 February 2011	16	CC	m	60	pes planovalgus	l	2	14 March 2011	good	79	130	1; 2	n
N = 72	24 May 1996	14 February 2011	14	CC	f	62	pes cavus	r	2	31 March 2011	good	63	63	n	n
N = 73	03 January 2000	28 April 2011	11	CC	f	52	clubfoot	r	2	10 June 2011	good	507	507	3; 4; 6	n
N = 74	19 December 1993	25 May 2011	17	CC	m	60	clubfoot	l	4	04 July 2011	good	58	58	n	n
N = 75	16 September 1999	15 June 2011	11	CC	m	34	pes cavus	r	2	25 July 2011	good	56	56	n	n
N = 76	23 April 2001	20 July 2011	10	CC	f	37	clubfoot	l	2	01 September 2011	pseudarthrosis	31	31	1; 2	n
N = 77	21 September 1997	20 September 2011	13	CC	m	50	clubfoot	l	2	07 November 2011	good	38	38	1	n
N = 78	17 February 1998	04 October 2011	13	CC	f	37	clubfoot	r	2	10 November 2011	good	159	159	n	n
N = 79	21 January 1997	01 December 2011	14	CC	m	101	clubfoot	l	2	05 January 2012	good	88	88	n	n
N = 80	13 February 2002	14 December 2011	9	CC	f	37	clubfoot	l	2	23 January 2012	good	210	238	n	n
N = 81	26 May 2004	05 January 2012	7	CC	m	15	pes planovalgus	l	3	07 February 2012	good	420	420	3; 4; 5	n
N = 82	25 February 2004	24 January 2012	7	CC	m	27	clubfoot	l	4	22 February 2012	good	197	197	1; 2	n
N = 83	02 April 1997	08 February 2012	14	CC	m	58	clubfoot	r	2	26 March 2012	good	85	85	n	n
N = 84	23 March 1997	01 March 2012	14	CC	m	74	pes cavus	r	4	16 April 2012	good	27	46	n	n
N = 85	13 August 2000	02 May 2012	11	CC	m	40	pes calcaneus	l	1	13 June 2012	good	32	32	n	n
N = 86	02 April 1996	08 May 2012	16	CC	m	40	pes cavus	l	2	22 June 2012	good	46	98	n	n
N = 87	13 February 2002	09 May 2012	10	CC	f	43	clubfoot	r	2	18 June 2012	good	170	170	n	n
N = 88	13 August 2000	13 June 2012	11	CC	m	35	pes planovalgus	r	2	20 July 2012	good	26	26	1	n
N = 89	19 May 2000	18 December 2012	12	CC	f	44	pes cavus	r	2	01 February 2013	good	26	26	n	n
N = 90	20 November 1999	19 March 2013	13	CC	m	38	clubfoot	r	2	02 May 2013	good	186	186	n	n
N = 91	05 September 2001	27 August 2013	11	CC	m	62	pes planovalgus	r	2	07 October 2014	good	119	119	n	n
N = 92	07 December 2003	11 November 2013	9	CC	m	20	pes planovalgus	l	3	12 December 2013	good	61	61	n	n
N = 93	12 June 1999	12 May 2014	14	CC	m	38	pes planovalgus	l	3	30 June 2014	good	183	183	n	n
N = 94	13 October 1997	01 July 2014	16	CC	m	130	pes planovalgus	r	2	14 August 2014	good	53	53	n	n
N = 95	05 September 2001	21 October 2014	13	CC	m	70	pes planovalgus	l	2	24 November 2014	good	59	59	n	hypothyroidism
N = 96	06 January 1997	03 December 2014	17	CC	m	84	clubfoot	r	2	15 January 2014	good	62	62	n	n
N = 97	25 August 2001	02 April 2015	13	CC	f	67	clubfoot	l	4	18 May 2015	good	189	189	n	n
N = 98	17 June 2003	23 June 2015	12	CC	f	54	pes planovalgus	l	2	03 August 2015	good	310	310	n	n
N = 99	20 March 2007	11 August 2015	8	CC	m	30	pes calcaneus	l	2	10 September 2015	good	47	47	n	n
N = 100	20 March 2007	17 August 2015	8	CC	m	30	pes calcaneus	r	2	14 September 2015	good	46	46	n	n
N = 101	06 November 2003	24 November 2015	12	CC	f	40	skewfoot	r	2	04 January 2015	good	31	31	n	n
N = 102	06 August 2002	16 December 2015	13	CC	m	43	pes cavus	l	2	27 January 2016	good	104	130	n	scoliosis
N = 103	24 July 1992	05 January 2010	17	MT1	m	50	pes cavus	l	1	15 February 2010	good	142	142	n	n
N = 104	24 July 1992	13 April 2010	17	MT1	m	51	pes cavus	r	1	26 May 2010	good	110	110	n	n
N = 105	17 February 1998	18 May 2010	12	MT1	f	35	clubfoot	l	2	05 July 2010	pseudarthrosis	231	231	1	n
N = 106	26 October 1996	03 January 2011	14	MT1	f	59	pes cavus	l	1	07 February 2011	good	41	63	1; 2	n
N = 107	09 August 1995	07 March 2011	15	MT1	m	38	skewfoot	l	2	21 April 2011	good	102	102	n	n
N = 108	21 September 1997	20 September 2011	13	MT1	m	50	clubfoot	l	1	07 November 2011	good	38	38	1	n
N = 109	23 March 1997	01 March 2012	14	MT1	m	74	pes cavus	r	1	16 April 2012	good	27	46	n	n
N = 110	02 April 1996	08 May 2012	16	MT1	m	40	pes cavus	l	1	22 June 2012	good	46	98	n	n
N = 111	19 May 2000	18 December 2012	12	MT1	f	44	pes cavus	r	2	01 February 2013	good	26	26	n	n
N = 112	20 December 1997	18 June 2013	15	MT1	m	100	clubfoot	l	2	25 July 2013	good	30	30	n	n
N = 113	28 September 1997	24 July 2013	15	MT1	f	53	hallux valgus	l	2	02 September 2013	good	27	29	n	n
N = 114	06 January 1997	03 December 2014	17	MT1	m	84	clubfoot	r	1	15 January 2014	good	62	62	n	n
N = 115	29 June 2002	19 August 2015	13	MT1	m	53	clubfoot	r	1	17 September 2015	good	198	198	n	n
N = 116	06 November 2003	24 November 2015	12	MT1	f	40	skewfoot	r	1	04 January 2015	good	31	31	n	n
N = 117	06 August 2002	16 December 2015	13	MT1	m	43	pes cavus	l	2	27 January 2016	good	104	130	n	scoliosis

Abbreviations: TN = Talonavicular; CC = Calcaneocuboidal; MT I = Metatarsale I; m = male; f = female; r = right; l = left; Complications: 1 = wound healing disorder; 2 = Antibiotics; 3 = relapse; 4 = Revision surgery; 5 = Chopart-AD; 6 = supramalleolar osteotomy.

**Table 3:** Overview of surgical procedures and indications.

Osteotomy and Surgical Technique	Indication
Evans osteotomy (lengthening osteotomy of the calcaneal neck) [18]	Planovalgus, skewfoot
Subtalar fusion (lateral or medial open or closed wedge)	Hindfoot varus/valgus with instability Severe bony fixed varus/valgus
Triple arthrodesis (Chopart’s and subtalar joint fusion)	Severe planovalgus, cavovarus with severe hindfoot varus, severe clubfoot (residual)
Lambrinudi procedure (triple fusion subtalar ventral-based wedge resection) [19]	(Bony) fixed hindfoot equinus
Inverse lambrinudi (additive triple fusion and subtalar dorsal-based wedge resection) [20]	Severe calcaneal foot
Chopart’s arthrodesis	Clubfoot, cavovarus foot
Grice procedure [21]	Planovalgus foot
Calcaneocuboid distraction fusion	Planovalgus foot

**Table 4:** Overview of the non-unions in this study.

Patient	Non-Union	Gender	Weight	Age	Underlying Condition	Number of K-wires	Diagnosis	Surgery	Other
N = 1	TN + CC	Male	93 kg	13 years	No	2	Clubfoot	Chopart-Arthrodesis	3 Revision surgery due to wound healing disorders
N = 2	TN + CC	Female	37 kg	10 years	No	4	Clubfoot relapse	Chopart-Arthrodesis	Antibiotics, Wound healing disorder
N = 3	TN	Male	27 kg	8 years	Respiratory disease	2	Clubfoot	Chopart-Arthrodesis	Previous surgery pantalare release
N = 4	MT I + CC	Female	35 kg	12 years	No	2	Clubfoot	Chopart-Arthrodesis + Correction MT I	Wound healing disorder

## Data Availability

The data presented in this study are available on request from the corresponding author. The data are not publicly available due to sensitive patient data.
